# Prognostic Factors and Markers in Non-Small Cell Lung Cancer: Recent Progress and Future Challenges

**DOI:** 10.3390/genes14101906

**Published:** 2023-10-04

**Authors:** Débora Dummer Meira, Maria Clara de Castro e Caetano, Matheus Correia Casotti, Aléxia Stefani Siqueira Zetum, André Felipe Monteiro Gonçalves, André Rodrigues Moreira, Augusto Henrique de Oliveira, Fellipe Pesente, Gabriel Mendonça Santana, Daniel de Almeida Duque, Gierleson Santos Cangussu Pereira, Giulia de Souza Cupertino de Castro, Isabele Pagani Pavan, João Pedro Sarcinelli Chagas, José Henrique Borges Bourguignon, Juliana Ribeiro de Oliveira, Karen Ruth Michio Barbosa, Lorena Souza Castro Altoé, Luana Santos Louro, Luiza Poppe Merigueti, Lyvia Neves Rebello Alves, Marlon Ramos Rosado Machado, Maria Luísa Rodrigues Oliveira Roque, Pedro Santana Prates, Sayuri Honorio de Paula Segáua, Taissa dos Santos Uchiya, Thomas Erik Santos Louro, Vinicius Eduardo Daleprane, Yasmin Moreto Guaitolini, Creuza Rachel Vicente, Raquel Silva dos Reis Trabach, Bruno Cancian de Araújo, Eldamária de Vargas Wolfgramm dos Santos, Flávia de Paula, Tiago José S. Lopes, Elizeu Fagundes de Carvalho, Iúri Drumond Louro

**Affiliations:** 1 Núcleo de Genética Humana e Molecular, Centro de Ciências Humanas e Naturais, Departamento de Ciências Biológicas, Universidade Federal do Espírito Santo (UFES), Vitória 29075-910, Brazilmatheus.c.casotti@gmail.com (M.C.C.);; 2 Centro de Ciências da Saúde, Curso de Medicina, Universidade Federal do Espírito Santo (UFES), Vitória 29090-040, Brazil; 3 Escola Superior de Ciências da Santa Casa de Misericórdia de Vitória (EMESCAM), Curso de Medicina, Vitória 29027-502, Brazil; 4 Departamento de Medicina Social, Universidade Federal do Espírito Santo, Vitória 29090-040, Brazil; 5 Department of Reproductive Biology, National Center for Child Health and Development Research Institute, Tokyo 157-8535, Japan; 6 Instituto de Biologia Roberto Alcântara Gomes (IBRAG), Universidade do Estado do Rio de Janeiro (UERJ), Rio de Janeiro 20551-030, Brazil

**Keywords:** lung cancer, NSCLC, prognostic markers, genotyping, *EGFR* mutations

## Abstract

Lung cancer is a highly aggressive neoplasm and, despite the development of recent therapies, tumor progression and recurrence following the initial response remains unsolved. Several questions remain unanswered about non-small cell lung cancer (NSCLC): (1) Which patients will actually benefit from therapy? (2) What are the predictive factors of response to MAbs and TKIs? (3) What are the best combination strategies with conventional treatments or new antineoplastic drugs? To answer these questions, an integrative literature review was carried out, searching articles in PUBMED, NCBI-PMC, Google Academic, and others. Here, we will examine the molecular genetics of lung cancer, emphasizing NSCLC, and delineate the primary categories of inhibitors based on their molecular targets, alongside the main treatment alternatives depending on the type of acquired resistance. We highlighted new therapies based on epigenetic information and a single-cell approach as a potential source of new biomarkers. The current and future of NSCLC management hinges upon genotyping correct prognostic markers, as well as on the evolution of precision medicine, which guarantees a tailored drug combination with precise targeting.

## 1. Introduction

Lung cancer has a high mortality rate and is the leading cause of death due to malignant neoplasms worldwide [1]. Incidence rates of this neoplasm are on the rise worldwide; nonetheless, the number of new cases is subject to regional variations and may either increase or decrease in a particular country [2]. Among the pathological subtypes of lung cancer, non-small cell lung cancer (NSCLC) is the most frequently diagnosed, with a prevalence of up to 85% in all cases [1,2,3,4]. NSCLC mainly comprises lung adenocarcinomas, squamous cell carcinoma, and giant cell carcinoma [3,4].

The elevated mortality rate associated with lung cancer is mainly due to common late diagnosis, given that only 2–20% of NSCLC patients survive beyond five years following diagnosis. In the last two decades, the use of targeted therapy in tumors with oncogenic mutations has considerably improved the treatment of advanced-stage NSCLC, with increased survival and better patient quality of life [1,4]. In the last few decades, there has been a growing interest in NSCLC oncogenic drive mutations [2,4].

Overexpression of the *EGFR* (epidermal growth factor receptor) gene is associated with higher tumor aggressiveness, decreased survival rate, and a higher number of metastases. Other genetic alterations are related to the development of NSCLC, such as changes in anaplastic lymphoma kinase (*ALK*), *ROS 1* (*ROS* proto-oncogene 1), *MET* (mesenchymal-epithelial transition factor), *RET* (*RET* proto-oncogene), *NTRK 1-3* (neurotrophic tyrosine kinase type 1–3), and *HER2* (human epidermal growth factor receptor 2) genes. These genetic alterations have led to the development of specific pathway therapies. Although targeted inhibitor therapy improves NSCLC patient prognosis, the response is usually short-lived as the majority of patients develop resistance within a year of the initial treatment [4].

In this review, we focus on oncogenic drive mutations and molecular target therapies of therapeutic antibodies and tyrosine kinase inhibitors, addressing the mechanism of action, resistance, and reversal of resistance in NSCLC.

## 2. Epidemiology and Molecular/Genetic Basis of Lung Cancer

According to data from over 180 countries in the year 2020, approximately 2,206,771 cases of lung cancer were diagnosed (11.4% of all cancers), resulting in an 18% mortality rate (1,796,144 patients) [2]. Non-small cell lung cancer is the most diagnosed subtype in the world, despite incidence peculiarities associated with social factors such as education and environmental conditions [2,5]. The incidence is high and is steadily increasing in the age group over 45 years, indicating the age target of screening tests [2]. The lack of reliable markers for initial-stage NSCLC tumors leads to late diagnoses and contributes to its high mortality rate [2]. Research has been conducted to explore miRNAs and other strategies’ potential as early diagnostic tools for this disease [2].

Lung cancer rates have been decreasing in developing countries. Despite smoking being the main risk factor, it is remarkable that over 10% of individuals with lung cancer are non-smokers, indicating other unknown risk factors [2]. For instance, in China, the high exposure to smoke from burning coal as fuel is a significant risk factor [2]. An analysis was conducted to investigate differences in *EGFR* mutations among various ethnic populations with lung adenocarcinomas. The results revealed a higher prevalence of *EGFR* mutations in Asians, with approximately 45% of adenocarcinomas exhibiting this alteration, and Latin Americans, with over 33% [6]. 

Moreover, US African Americans have been observed to have divergent cancer rates compared to other populations, with higher mortality rates and shorter survival times. While socioeconomic factors may contribute to these disparities, evidence suggests that differences in tumor biology may also play an important role. However, the precise relationship between tumor biology and race/ethnicity has yet to be fully elucidated [7].

Cigarette smoking is a well-known risk factor for the development of neoplasms, as it induces mutations in genes that play a role in cellular signaling pathways and regulation of the cell cycle, including *KRAS* (Kirsten rat sarcoma virus) and *TP53* (Tumor protein p53). Exposure to pollutants is also associated with an increased risk of cancer development, as it can alter cellular processes and damage genetic material. Particulate matter with a diameter of less than 2.5 μm (PM), which contains benzopyrenes and polycyclic aromatic hydrocarbons, is a pollutant that induces DNA damage, inflammation, and angiogenesis, creating an environment that promotes tumor development [5,8]. For instance, there is evidence that PM exposure is associated with mutations in the *TP53* gene [9].

The NSCLC is characterized by genetic heterogeneity, which poses a major challenge for developing effective therapies. To achieve successful treatment, it is crucial to identify driver mutations that cause neoplastic transformation. However, the presence of multiple mutant variants results in a range of metabolic phenotypes, making it difficult to standardize individualized treatment methods. Therefore, a universal inhibitor for NSCLC is unlikely to be developed in the near future [2].

Lung epithelial cells are prone to developing mutations that can activate oncogenes or cause the loss of suppressor gene function, thus creating a favorable environment for the formation of lung neoplasms [10]. Alterations in tumor suppressor genes can negatively impact the efficacy of therapeutic interventions. For instance, in the context of lung cancer, drugs capable of inhibiting abnormal tumor suppressor function have been employed, such as a combination of transforming growth factor-β-activated kinase 1 (TAK1) inhibitor and MEK1/2 inhibitor [10]. Notable molecular alterations associated with lung cancer include *KRAS*, *EGFR*, *ALK*, *TP53*, and *TAK1-NF-kB*, which will be described subsequently.

### 2.1. KRAS

*KRAS* is the most common oncogene in hereditary lung cancer, participating in up to 25% of adenocarcinomas and 3% of squamous cell carcinomas. The most common mutations involve codons 12 and 13 [11].

Knowing the molecular mechanisms and the proteins involved in their expression patterns is fundamental to discovering new treatment options. Approved drugs already exist for some mutant proteins: *EGFR*, *ALK*, *ROS1*, *MET*, *RET*, *NTRK*, and *RAF* (rapidly accelerated fibrosarcoma). However, the K-RAS protein remains a challenge for lung cancer treatment [2].

K-RAS belongs to the RAS g-protein family, along with two other proteins, N-RAS and H-RAS. It represents a major difficulty to oncological treatment, as its mutation induces a variety of mechanisms and phenotypes that cannot be suppressed with only one inhibitor [2]. In the last few years, advances have motivated the projection of drugs for K-RAS [5]. *KRAS-G12A* is a common mutation in NSCLC-like lung tumors, and it is the main target of new research in oncology [5].

Although there is conflicting information in the literature about the prognostic and predictive value of KRAS mutations, they are supposedly related to poor survival and a negative response to EGFR receptor tyrosine kinase inhibitors. However, they also are positive predictors of response to ICIs (immune checkpoint inhibitors) [5].

### 2.2. EGFR

The epidermal growth factor receptor (*EGFR*) or *ERBB1* is a transmembrane receptor belonging to the tyrosine kinase family, expressed on the membrane of epithelial cells. In individuals with lung adenocarcinoma, up to 20% exhibit mutations in the *ERBB1* gene, which encodes the EGFR protein. This protein participates in signaling pathways that regulate the cell cycle; however, its functionality becomes aberrant in neoplastic cells, causing dysregulated signaling and promoting accelerated and aberrant proliferation of tumor cells, while inhibiting apoptotic pathways [2].

Among resistance factors, the aryl hydrocarbon receptor (*AhR*) has its frequency increased in NSCLC, and it is related to *EGFR* resistance. It acts on the recruitment of Src, leading to its phosphorylation by Jak2 (Janus kinase 2). Therefore, it preserves signaling pathways promoted by *EGFR*, such as PI3K/Akt (Phosphatidylinositol 3-kinase/protein kinase B) and MEK/Erk (Mitogen-activated ERK kinase). In addition, *AhR* acts in mechanisms that lead to oncogenesis, such as alterations in the cell cycle and evasion of programmed cell death [12].

The *CXCR7* (C-X-C chemokine receptor type 7) gene belongs to the G-protein-coupled receptor family and can activate signaling pathways, such as *PI3K* and *MAPK*, that are associated with *EGFR*. The influence of *CXCL12* (C-X-C Motif Chemokine Ligand 12) leads to homodimerization of *CXCR7* by associating with β-arrestin2, or heterodimerization with *CXCR4* (C-X-C chemokine receptor type 4), thereby activating cell signaling. An increase in *CXCR7* expression can lead to resistance to tyrosine kinase inhibitors and contribute to the establishment of the tumor microenvironment, stimulating processes such as metastasis, angiogenesis, and cell division. *CXCR7* is overexpressed in non-small cell carcinomas, with or without *T790M* mutations [13].

*BZW1* (basic leucine zipper and W2 domains 1) is a gene that belongs to the bZIP (basic leucine zipper) family of transcription factors. Its domains are related to other important factors, such as ATF (activating transcription factor), JUN (Transcription factor Jun), CREB (cAMP-response element binding protein), and NRF2 (nuclear factor erythroid 2-related factor 2). In lung neoplasms, the expression of *BZW1* may be linked to tumor migration and plays a role in metastasis formation by interacting with *EGFR* [14].

In this context, *T790M* mutations (which can also be acquired) impact the affinity of the *EGFR* binding site for ATP. Thus, it increases its interaction and signaling, possibly leading to acquired resistance to tyrosine kinase inhibitors. Several new inhibitors have been studied aiming to reverse tumor resistance [15].

### 2.3. ALK

The ALK (anaplastic lymphoma kinase) receptor is involved in several processes related to the cell life cycle, although its exact function remains undefined [2]. Abnormal bound protein production occurs when the *ALK* gene fuses with another gene, due to its location at a common chromosomal translocation hotspot. Such a mechanism is seen in adenocarcinomas with an incidence of 5–6% [2,16].

The first *ALK* rearrangement in NSCLC was identified between ALK and the EM4 protein, which results in a large inversion or translocation and stimulates oncogenic activity. The *EML4-ALK* rearrangement appears to be mutually exclusive with *EGFR* and *KRAS* mutations [16].

### 2.4. TP53

In NSCLC patients, *TP53* is the most frequently mutated gene, encoding the cell cycle regulatory protein p53. It is known as the guardian of the genome due to being the main gene responsible for the G1/S cell cycle checkpoint [5]. *TP53* loss of heterozygosity (LOH) results in cells that only express the mutant, causing the loss of p53 protein activity [2].

Several studies aimed to assess the relationship between *TP53* mutations with a tumor mutational burden and benefit with ICIs [8]. *TP53* mutation status is currently related to overall survival in NSCLC patients who undergo immunotherapy with PD-1/PDL-1 blockers [8]. 

Li-Fraumeni syndrome is a rare hereditary cancer susceptibility syndrome associated with a mutation of the *TP53* tumor-suppressor gene. Those patients are more likely to develop sarcoma, brain tumors, leukemia, lung adenocarcinoma, among others. Current guidelines state that these patients should undergo an annual magnetic resonance imaging scan to screen for these different cancer types [17].

### 2.5. TAK1-NF-kB

The *CLU* gene encodes the clusterin glycoprotein, which assists the protein folding process similarly to heat-shock proteins. It is related to cellular processes, such as the regulation of cell division, the process of programmed cell death, and the repair of genetic material. It plays an essential role in blocking TAK1-NF-kB (nuclear factor kappa B) signaling, by the inhibition of TGFBR1 (transforming growth factor-β receptor type 1). Therefore, it will not recruit TRAF6 (tumor necrosis factor receptor-associated factor 6)/TAB2 (TGF-β activated kinase 1/MAP3K7-binding protein 2)/TAK1 complex. The latter nuclear factor activates NF-kB, which is related to the transcriptional mechanism of IL-1 (Interleukin 1), TNF (tumor necrosis factor), and JUN. Thus, alterations in the *CLU* gene can lead to the development of cancer, including NSCLC [10].

## 3. Molecular Targeted Therapy in Lung Cancer

A major issue associated with treatment using specific inhibitors is tumor resistance. We will describe below the main classes of inhibitors (according to their molecular targets), as well as the main treatment options according to the acquired resistance.

### 3.1. KRAS

Although there are currently no treatments capable of fully addressing *KRAS* mutations, AMG510 is a promising molecule capable of selectively inhibiting *KRAS G12C*, by binding to guanosine diphosphate (GDP). In a phase 1 clinical trial evaluating AMG510 in ten adult patients with locally advanced or metastatic *KRAS G12C*-positive tumors, five patients showed partial response to treatment with an overall response rate (ORR) of approximately 50%, four patients had stable disease, while one patient had disease progression, and the most common adverse effects were loss of appetite and diarrhea [18].

Additionally, MRTX849 has shown to be another highly selective inhibitor of *KRAS G12C*, relevant in the treatment of advanced malignancies. A phase I/II clinical trial was conducted in which 17 patients received MRTX849 at doses ranging from 150 mg to 1200 mg daily. Out of ten NSCLC patients treated, six were evaluable for response and three showed a partial response with an ORR of approximately 50%. The most common adverse reactions consisted of decreased appetite, diarrhea, nausea, vomiting, elevated liver enzymes, and increased creatinine, as well as documented grade 3 toxicities, including fatigue, decreased appetite, and dyspnea [19]. There are currently no conclusive comprehensive studies available on the combined application of AMG510 and MRTX849.

### 3.2. EGFR TKIs (EGFR Tyrosine-Kinase Inhibitors)

The mutations associated with NSCLC occur specifically within exons 18–21, with approximately 90% of these mutations being exon 19 deletions or exon 21-point mutations L858R. Consequently, these mutations demonstrate specific sensitivity to *EGFR* TKIs, whereas *EGFR* mutations in exon 18 and exon 20 are generally less sensitive to *EGFR* TKIs [20].

Several treatments approved by the Food and Drug Administration (FDA) for patients with metastatic NSCLC whose tumors harbored the previously mentioned mutations include gefitinib, erlotinib, afatinib, dacomitinib, and osimertinib. In addition, afatinib has been shown to be specifically effective against NSCLC from uncommon *EGFR* mutations, especially Gly719Xaa, Leu861Gln, and Ser768Ile, but less effective against other mutations [20].

In this context, a phase 3 clinical trial revealed that osimertinib was able to prolong the survival of progression-free patients to 18.9 months compared to 10.2 months with gefitinib or erlotinib treatments. Although all three agents showed similar response rates of around 80%, the duration of response was significantly longer for osimertinib. Moreover, grade 3 or 4 adverse events were reported less frequently with osimertinib. Finally, results from the phase 3 clinical trial FLAURA showed a median overall survival of 38.6 months in patients who received osimertinib compared to 31.8 months in patients who received gefitinib/erlotinib [21].

Patients with *EGFR/HER2* exon 20 mutations represent approximately 10% of all cases of NSCLC caused by *EGFR* mutation, as these mutations are known to confer primary resistance to TKIs [22].

Recent studies have described the usefulness of two new agents for this subtype of NSCLC-TAK-788 and poziotinib. TAK-788 is an investigational TKI capable of inhibiting *EGFR* and *HER2* receptors. In a phase I/II clinical trial, 101 patients received TAK-788 at doses ranging from 5 to 180 mg, with the recommended dose for phase II being 160 mg. Efficacy was favorable in 24 patients, with 23 patients showing a decrease in target lesions with a median percentage variation of 32.6%. Patients who received a dosage of 160 mg had an ORR of 54%, and adverse effects were similar to other *EGFR* TKIs [22,23].

Acquired *MET* amplification constitutes the second most common resistance mechanism and occurs in approximately 20% of patients who experienced disease progression after treatment with gefitinib or erlotinib and may occur in up to 30% of patients treated with osimertinib [24,25,26].

Other noteworthy resistance mechanisms consist of *HER2* amplification, activation of the RAS/MAPK/PI3K pathway, cell cycle alteration, and transformation into small cell lung cancer [24,25,26].

An issue associated with *EGFR* TKI treatment is the inevitable return of disease progression after the initial response. About half of the patients who present disease progression after gefitinib, erlotinib, or afatinib develop resistance related to *EGFR T790M*. To overcome such resistance, the administration of osimertinib is indicated, and if disease progression occurs after osimertinib, highly heterogeneous resistance development is observed through *EGFR*-dependent and independent mechanisms. For such patients who progressed after osimertinib, it is recognized that there is still no FDA-approved targeted therapy [26].

### 3.3. ALK

For patients with NSCLC harboring an *ALK* (anaplastic lymphoma kinase) rearrangement mutation, characterized by inversion of the *EML4* and *ALK* genes on chromosome 2 that typically occurs in young, non-smoking individuals, molecular targeted therapy using *ALK* TKIs can also be used to inhibit expression of this newly formed oncogene [20].

Crizotinib is used as one of the first targeted therapies for *ALK*-positive NSCLC (first-generation therapy), which shows a longer progression-free survival (PFS) when compared to chemotherapy, with a PFS of 10.9 months for crizotinib compared to 7.0 months for chemotherapy. However, in addition to its low penetration into the central nervous system, its use consequently leads to the development of resistance. Therefore, new generations of drugs have been developed with the goal of achieving greater potency in treatment and less resistance [27].

Alectinib is a second-generation inhibitor used as a first-line treatment in advanced NSCLC, as it has a higher survival rate compared to crizotinib, with 68.4% compared to 48.7% over a period of 12 months. In addition, the PFS achieved by alectinib was 34.8 months, also higher than that of crizotinib. Another second-generation *ALK* inhibitor is brigatinib, which also showed better efficacy when compared to crizotinib, with a 12-month survival rate of 67%. Finally, there is ceritinib, which, although not directly compared to crizotinib, showed better treatment outcomes [28,29,30,31].

Lorlatinib is a third-generation *ALK* TKI used in cases of *ALK*-positive NSCLC that has progressed with the use of alectinib or ceritinib, or crizotinib and at least another *ALK* inhibitor. In this therapy, the ORR was shown to be 47%, and in 7% of tested patients serious side effects occurred, with the most common adverse effects being hypercholesterolemia, hypertriglyceridemia, edema, and peripheral neuropathy [32].

### 3.4. TP53

Currently, no drug used for *TP53* mutations is available on the market. However, a combination of *EGFR* TKIs with anti-angiogenic agents, chemotherapy, or other drugs can be used. There are clinical trials using p53 protein activators such as ARP-246, seeking potential methods to restore its tumor suppression function [33].

A recent phase III randomized study showed that the combination of gefitinib, an *EGFR* inhibitor, with carboplatin and pemetrexed, which are chemotherapeutic agents, resulted in an increased PFS in patients with NSCLC with *EGFR* mutations when compared to gefitinib alone, with an increase from 11.2 months to 20.9 months. Currently, although there is no study that specifically associates *EGFR* TKIs with chemotherapy in advanced NSCLC with combined *EGFR* and *TP53* mutations, studies are being conducted comparing monotherapy with *EGFR* inhibitors to the combined therapy of *EGFR* and chemotherapy [33,34].

Therefore, in the combination of *EGFR* TKIs and antiangiogenic drugs, studies have shown significant improvement in PFS compared to the use of *EGFR* inhibitors alone. In this case, it was observed that the combination of ramucirumab, an antiangiogenic agent, with erlotinib in patients with combined *EGFR* and *TP53* mutations led to a better PFS than in the treatment with erlotinib alone. Additionally, another study by Cheng Y et al. showed that the use of bevacizumab, a VEGF inhibitor, combined with *EGFR* TKI resulted in an increase from 9.7 months to 14 months in PFS in patients with *EGFR* and *TP53* co-mutations compared to the use of *EGFR* TKI alone [35,36].

No PFS improvement was observed when using anti-PD-1 (programmed death 1 protein) and anti-PD-L1 (programmed death 1 ligand) antibodies in patients with *EGFR* mutations. Thus, according to the current guidelines of the National Comprehensive Cancer Network (NCCN), immunotherapy is not recommended for the treatment of NSCLC patients together with *EGFR* inhibitors [37,38,39].

## 4. Clinical Trials of Therapeutic Antibodies and TKIS

In the last decade, several biomarkers have been explored for the development of drugs aimed at treating NSCLC, many of which have been approved by the FDA, as described above [20]. Monoclonal antibodies against PD-1 and PD-L1, such as pembrolizumab, stand out. However, there are several other classes of drugs evaluated in phase I, II, and III clinical trials, such as anti-TROP2 mAbs, TKIs, *ALK* arrangement inhibitors, and *MEK* inhibitors (Mitogen-activated protein kinase kinase). We will describe below the main drugs developed in recent years for the treatment of lung cancer, as well as information on their respective clinical studies and their importance for NSCLC treatment.

### 4.1. Nivolumab

Humanized monoclonal antibody immunoglobulin G4 against PD-1’s approval was granted in 2015 based on a randomized phase III study that compared it with docetaxel in patients with advanced NSCLC [34]. In this study, patients receiving nivolumab had a median overall survival of 9.2 months (95% CI: 7.3–13.3) compared to 6 months for docetaxel (95% CI: 5.1–7.3), with a hazard ratio (HR) of 0.59 (95% confidence interval (CI): 0.44–0.79; *p* < 0.001); and the one-year overall survival with nivolumab was 42%, while that with docetaxel was 24%. Adverse effects of nivolumab were also less frequent than those observed with docetaxel (58% and 86%, respectively), which helped patients’ continuity in therapy. 

Studies have shown a benefit by combining nivolumab with ipilimumab, a humanized antibody against cytotoxic T-lymphocyte-associated antigen-4 (CTLA-4). The first to demonstrate this was CheckMate-012, a phase I study in which patients were assigned to three different therapeutic regimens: nivolumab 1 mg/kg every 2 weeks plus ipilimumab 1 mg/kg every 6 weeks, nivolumab 3 mg/kg every 2 weeks plus ipilimumab 1 mg/kg every 12 weeks, and nivolumab 3 mg/kg every 2 weeks plus ipilimumab 1 mg/kg every 6 weeks. It was then observed that the groups that received ipilimumab every 6 weeks had better treatment outcomes [40]. 

Based on this, the CheckMate 227 study was conducted, which compared patients undergoing only standard chemotherapy and patients using a combination of nivolumab and ipilimumab. It was observed that for the subgroup with PD-L1 ≥ 1%, the median overall survival was 17.2 months in the group receiving the immunotherapy combination, compared to 12.2 months in the group receiving only chemotherapy [41]. In addition, the duration of response was significantly longer in the group that received nivolumab plus ipilimumab, being 23.2 months compared to 6.2 months for the conventional chemotherapy treatment [40].

### 4.2. Pembrolizumab

Pembrolizuma is a humanized monoclonal antibody IgG4 against PD-1. Its first evaluation for treatment of NSCLC occurred in a phase 1 study involving both previously treated and untreated patients at three different doses: 2 mg/kg every 3 weeks, 10 mg/kg every 3 weeks, or 10 mg/kg every 2 weeks (KEYNOTE-001). The overall response rate was similar between different dosages, therapeutic schedules, and histological types of tumors, corresponding to 19.4% (95% CI: 16.0–23.2). The median duration of response was 12.5 months, with 84.4% of patients showing no disease progression. The median overall survival was 12 months [40]. Interestingly, although immunotherapies have low effectiveness in patients whose cancer etiology derives from genetic abnormalities, pembrolizumab has relatively high efficacy in patients with *EGFR* mutation [42]. 

In general, studies show that the most reliable biomarker currently to identify the effectiveness of therapy with pembrolizumab and other PD-1 inhibitors is PD-L1 expression [43]. As side effects, dermatological reactions, fatigue, itching, nausea, diarrhea, loss of appetite, asthenia, hypo- and hyperthyroidism, and arthralgia can be mentioned. In addition, there are reports of hepato- and nephrotoxicity, pneumonitis, neutropenia, and rare cases of myasthenia gravis [44]. 

### 4.3. Durvalumab

Humanized monoclonal antibody IgG1 against PD-L1 was approved for the treatment of urothelial carcinoma, showing efficacy in maintenance therapy of unresectable NSCLC after chemotherapy (PACIFIC study). This study compared the administration of 10 mg/kg durvalumab and placebo for 12 months. The PFS in the group that received the drug was 16.8 months, compared to 5.6 months in the placebo group. Adverse effects had the same incidence rate in both groups [40].

### 4.4. Sacituzumab Govitecan

Recently drugs target trophoblast cell surface antigen 2 (TROP2), a calcium signal transducer associated with tumor-2 encoded by the *TACSTD2* gene. This transmembrane glycoprotein is increased in various epithelial tumors, including NSCLC. Sacituzumab Govitecan was tested in a multicenter, phase 2 clinical trial (NCT03964727) in participants with metastatic solid tumors. The average objective response rate was 17%, and the median duration of tumor response was 3.8 months (1.8–11.6 months). The median progression-free survival was 5.2 months (95% CI: 3.2–7.1 months), and the median overall survival was 9.5 months (95% CI: 5.9–16.7 months) [45].

### 4.5. Datopotamab Deruxtecan

Another drug targeting *TROP2* is Datopotamab Deruxtecan, a humanized monoclonal antibody against *TROP2* associated with Deruxtecan, a topoisomerase I inhibitor. It was evaluated in a phase I clinical trial called TROPION-PanTumor01, in which patients with NSCLC, triple-negative breast cancer (TNBC), and HR-positive breast cancer were evaluated. In the allocation of NSCLC patients treated with Datopotamab Deruxtecan, 159 patients were divided into three groups, in which they received doses of 4, 6, or 8 mg/kg every 3 weeks. The disease control rate was 73% for the 4 mg/kg dose, 67% for the 6 mg/kg dose, and 80% for the 8 mg/kg dose. The median progression-free survival was 4.3, 8.2, and 5.4 months, respectively. Side effects were more frequently observed in the groups with higher doses [45].

### 4.6. Afatinib

Afatinib is a second-generation tyrosine kinase inhibitor that reversibly inhibits the ErbB/HER receptor family, including *EGFR* and *ERBB2*. Its efficacy was initially tested by the Lux-Lung 3 study, in which patients who received initial therapy with afatinib were compared with those who received standard chemotherapy. The first group had a progression-free survival of 11.1 months, while the second had 6.9 months [46].

### 4.7. Gefitinib

Gefitinib is a first-generation *EGFR* TKI. It was initially tested in a phase 3 IPASS study, in which gefitinib therapy was compared to standard chemotherapy. Patients with *EGFR* mutations who received gefitinib had a longer PFS than the other group, an increased response rate (71.2% vs. 47.3%), and fewer side effects (e.g., neutropenia) compared to carboplatin/paclitaxel [47]. 

### 4.8. Erlotinib

Erlotinib is a first-generation *EGFR* TKI. The first study to test its efficacy in NSCLC was the EUTARC, a phase 3 trial in which the drug was compared to standard chemotherapy. The group receiving the TKI had a progression-free survival of 9.7 months, compared to 5.2 months in the chemotherapy group. In addition, adverse events were lower in the group exposed to erlotinib [48].

### 4.9. Dacomitinib

Dacomitinib is a second-generation TKI that irreversibly inhibits ErbB/HER receptors (*EGFR*, *HER1*, *HER2*, and *HER4*). It was tested in the phase 3 study ARCHER 1050, in which it was compared to gefitinib in *EGFR*-positive patients with metastatic NSCLC. The group treated with dacomitinib had a PFS of 14.7 months, while the group treated with gefitinib had a PFS of 9.2 months. However, patients in the first group experienced more frequent severe side effects than the second group (9% versus 4%) [49,50].

### 4.10. Osimertinib

Osimertinib is a third-generation oral TKI that inhibits common (exon 19 deletion and L858R) and uncommon (S768I, L861Q, and/or G719X) *EGFR* mutations. Its approval was based on the FLAURA study, which compared osimertinib to erlotinib or gefitinib in patients with metastatic NSCLC and *EGFR* mutations. The group treated with osimertinib had a longer PFS than the group treated with the other drugs (18.9 versus 10.2 months), as well as a longer median duration of response (17.2 versus 8.5 months). Serious adverse effects were also less frequently observed in this group (34% versus 45%) [51].

### 4.11. ALK Inhibitors

Regarding *ALK* rearrangement inhibitors, the first drug to be approved was crizotinib, which showed a significantly longer PFS compared to chemotherapy. However, due to consistently developed resistance to this drug, the preferred first-line treatment for *ALK*-positive cancers is alectinib. The two drugs were compared in the ALEX trial. In addition to having better tolerability, patients treated with alectinib showed a longer median PFS (34.8 months versus 10.9 for crizotinib) and a higher 12-month survival rate (68.4% compared to 48.7%) [20,52].

Another new-generation *ALK* inhibitor is brigatinib, whose one-year PFS was estimated at 67%, a considerable improvement compared to crizotinib’s 43%. The ORR was also higher, corresponding to 71% versus 60%. The most significant advantage is the intracranial response rate, evaluated at 78% for brigatinib and 29% for crizotinib. Certinib has also proven to be more potent than crizotinib and more effective in patients with progression on this drug, although they were not directly compared in the first-line context [20,52].

Finally, lorlatinib is a third-generation *ALK* and *ROS1* inhibitor. In a phase II clinical trial, its ORR and objective intracranial response were evaluated at 47% and 63%, respectively. The study was conducted in NSCLC *ALK*-positive patients who no longer had desirable responses to one or more drugs in this class. A range of side effects were observed, including hypercholesterolemia, hypertriglyceridemia, edema, and peripheral neuropathy [20,52].

### 4.12. MEK Inhibitors

*MEK* inhibitor drugs are in various phases of clinical trials. Trametinib, has been approved for use in combination with dabrafenib, a *BRAF* inhibitor, for BRAF^V600E^ NSCLC treatment. In the phase 2 clinical trial that led to its approval, 36 patients were treated with both drugs. The median duration of response was 10.4 months, the PFS was 10.9 months, and the ORR was 64% in patients previously treated with chemotherapy, with untreated patients showing similar responses (9 months, 9.7 months, and 63%, respectively). All patients experienced one or more side effects, the main ones being pyrexia, elevated alanine aminotransferase (ALT), hypertension, and vomiting. Three patients experienced more severe adverse effects, such as a reduction in cardiac ejection fraction [53]. 

Selumetinib was tested in combination with docetaxel for NSCLC treatment in the SELECT-2 study. There was an improvement in the ORR, which went from 14% with docetaxel alone to 33% with the drug combination. However, this was not reflected in the PFS, which was 4.3 and 4.2 months in the respective groups. There were no new safety risks to patients, and drug tolerability corresponded to what was already determined in the literature. In summary, there are promising studies and clinical trials regarding the use of *MEK* inhibitors for lung cancer treatment. However, resistance is often observed and new ways to improve their efficacy are needed, possibly through combination treatments [54]. 

**Figure 1:**
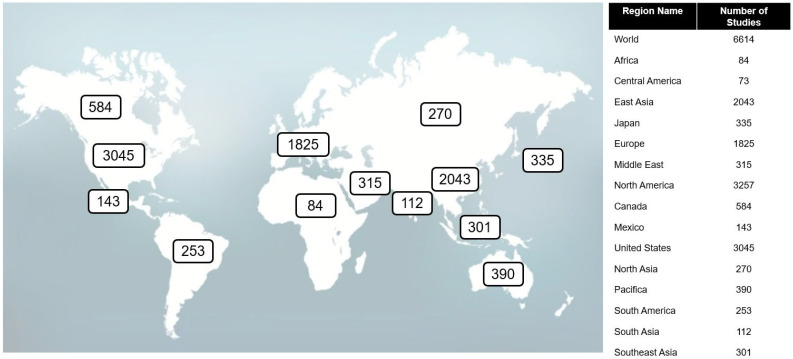
Global distribution of NSCLC clinical trials. Studies in Europe correspond to 27.6% and the United States correspond to 46% of all clinical trials. Labels give the exact number of studies located in different regions. Studies with no location are not included in the counts or on the map, and studies with multiple locations are included in all corresponding regions. Adapted from https://clinicaltrials.gov, accessed on 20 July 2023.

We have searched https://clinicaltrials.gov (accessed on 20 July 2023) for the term NSCLC, and 6614 studies were found. Figure 1 shows the distribution of clinical trials around the globe (Asia, Europe, and the United States), and the most relevant trials are shown in Table 1.

## 5. Inhibitors: Mechanism of Action

### 5.1. ALK Inhibitors

The ALK rearrangement, a fusion of ALK to EML4 genes, is found in approximately 5% of patients [30], who are generally young, non-smokers, and poor responders to EGFR inhibitors [55]. This rearrangement leads to oncogenic activity in vitro and in vivo [56], suggesting that upregulated tyrosine kinase activity directly impacts the tumor environment. ALK inhibitors such as alectinib and ceritinib operate by blocking this kinase, so patients who have an ALK-EML4 mutation benefit from this therapy [57]. Furthermore, this translocation is found in 1–2% of patients, a group for which crizotinib is the first line of treatment [58,59].

### 5.2. BRAF^V600E^ Inhibitors

Mutations in *BRAF*, which encodes serine/threonine kinase in the RAS-MAPK signaling pathway, are present in approximately 2–4% of NSCLC, mainly in lung adenocarcinoma, and the V600E corresponds to approximately 30% of them [60], in which case the first line of treatment is vemurafenib and dabrafenib, which inhibit *BRAF* type I serine/threonine kinase by binding to its active site with greater affinity for the *BRAF V600E* mutation, not affecting other kinases [61].

### 5.3. NTRK Fusion Inhibitors

The *NTRK* gene mutation (neurotrophic tyrosine kinase) is found in approximately 1% of lung cancer patients [62]. This gene family encodes tropomyosin receptor kinases (TRKA, TRKB, and TRKC), and fused *NTRK* genes (*NTRK1*, *NTRK2*, *NTRK3*) translate a TRK fusion protein that activates tyrosine kinases and constitutively stimulates the growth of tumor cells [63]. *NTRK* inhibitors larotrectinib and entrectinib are the first line of treatment for patients with this mutation and act by blocking TRK protein at the ATP binding site [64].

### 5.4. EFGR Inhibitors

The most prevalent *EGFR* mutations found in NSCLC are the following: exon 19 deletions (45%); exon 21 L858R point mutation (40%); exon 19 insertions, such as S768I, L861Q, G719X (in up to 10% of cases), and, rarely, exon 20 insertions (approximately 5% of cases) [65,66,67].

The two most common mutations respond well to tyrosine kinase inhibitors (TKI) [65,66,67]. First-generation inhibitors (erlotinib and gefitinib) bind reversibly to *EGFR* tyrosine kinase; second-generation ones (afatinib and dacomitinib) bind irreversibly, being more potent; and third-generation ones (osimertinib) have fewer adverse effects and are also active against tumors with T790M mutations [55]. 

However, tumors with exon 20 insertions do not respond well to TKIs, requiring the use of amivantamab-vmjw, a biospecific humanized antibody that acts on *EGFR* and *MET* receptors and possesses direct activity on cellular immunity [68]. Another drug used is mobocertinib, an oral TKI that selectively inhibits several exon 20 insertion mutations [69].

### 5.5. RET Rearrangements Inhibitors

*RET* (Rearranged during transfection) gene fusions are found in up to 2% of lung cancers [70]. The *RET* gene is an oncogenic driver encoding a receptor tyrosine kinase, which forms heterogeneous ternary complexes that induce autophosphorylation of *RET* TKI domains, leading to the activation of downstream signaling pathways responsible for cell proliferation, growth, differentiation, and survival, such as RAS/MAPK, PI3K/AKT, PKC, and JAK-STAT [70]. The recommended first-line drugs are selpercatinib, pralsetinib, both selective *RET* domain inhibitors, and cabozatinib, a multi-kinase inhibitor [71].

### 5.6. CD73-Targeted Therapy

CD73 (ecto-5′-nucleotidase anchored to glycosylphosphatidylinositol) is essential for the generation of extracellular adenosine from 5′-adenosine monophosphate (5′-AMP), being expressed by different cancer types, causing tumor growth, metastasis, and, in some cases, treatment resistance, by immune evasion of tumor cells, leading to adenosine accumulation, which interferes with NK cells’ cytotoxic ability [72]. CD73-targeted therapies, such as chimeric NK cells, show promise [73].

### 5.7. VEGF (Vascular Endothelial Growth Factor) Inhibitors

The VEGF receptor is critical in oncogenesis, acting on vascular proliferation and promoting an immunosuppressed tumor environment, which occurs by suppressing antigen presentation and stimulating regulatory T-cell activity [74]. Therapies targeting the VEGF/VEGF receptor (VEGFR) pathway, including neutralizing antibodies to VEGF or VEGFR and receptor tyrosine kinase inhibitors, have been proven effective. The first antiangiogenic antibody was bevacizumab, which inhibits neovascularization by binding to VEGF-A and blocking the interaction of VEGF-A with VEGFR, preventing VEGF signaling activation [75].

### 5.8. Anti-CLDN18.2

Found in tight junctions, CLDN18.2 (claudin18.2) is usually aberrantly expressed in cancer. In addition to being a prognostic biomarker, it is an important target of immunotherapy. Zolbetuximab tightly binds to CLDN18.2 protein without compromising other claudins, resulting in tumor cell lysis [76].

### 5.9. Anti-MARCO and Anti-IL37R

An accumulation of TAM (anti-inflammatory tumor-associated macrophages) is associated with worse prognosis and resistance, including MARCO (macrophage receptor with collagenous structure). Consequently, its increase is linked to a proliferation of regulatory T cells and effector T cells, in addition to a decrease in

NK cells and anti-inflammatory cytokine IL37 release. Cancerogenic cells have the ability to create an immunosuppressive environment by polarizing macrophages to express MARCO and release IL37. Thus, the MARCO or IL37 receptor (IL37R) is an important target for antibody or IL37 CRISPR, which would result in the repolarization of TAMs, and the consequent recovery of cytolytic and antitumor activity of NK and T cells [77].

### 5.10. KRAS Inhibitors

KRAS is a G protein with GTPase activity that acts in the MAP/ERK metabolic pathway. Point mutations in the *KRAS* gene usually occur in codon 12, the most common being the p.G12C mutation [78]. Patients with this mutation may be treated with sotorasib, an oral TKI, that is quite useful in patients with metastatic lung cancer after the previous use of chemotherapy regimens [79].

### 5.11. MET Inhibitors

C-MET is the receptor for HGF (hepatocyte growth factor) and a tyrosine-kinase receptor involved in cell survival and proliferation. Oncogenic alterations in the *MET* gene include METex14 skipping mutations, gene amplification or gain in copy number, and overexpression of the MET protein [80]. The immunotherapies used are capmatinib, crizotinib, and tepotinib, which are oral TKIs and act as selective inhibitors of *MET* oncogenic alterations [81,82,83].

### 5.12. ERBB2 (HER2) Inhibitors

Mutations in exon 20 of the *ERBB2* gene (*HER2*) occur in approximately 3% of patients with advanced non-small cell lung cancer [84]. To treat these tumors, two monoclonal drug-antibody conjugates are used: ado-trastuzumab emtansine and fam-trastuzumab deruxtecan-nxki, which act as selective inhibitors of these mutations [85,86].

### 5.13. Anti-PD-1/Anti-PD-L1 Antibodies

Cancer immunity is regulated by numerous inhibitory or stimulatory signals, which are collectively called immune checkpoints. Among these checkpoints are PD-1 and PD-L1. PD-1 is an inhibitory immune regulator expressed on active cytotoxic T cells. 

The ability of cytotoxic T lymphocytes to kill tumor cells is downregulated when PD1 binds to PD-L1. Tumor cells expressing PD-L1 can evade an immune attack by cytotoxic T cells and survive (adaptive resistance). Therefore, blocking these inhibitory molecules with immune checkpoint inhibitors (ICI) may restore the immune ability to kill tumor cells [87]. In fact, ICIs remove T cell activation blockage caused by antigens expressed by tumor cells, allowing T cells to mount an antitumor response by overriding the normal regulatory mechanisms of cell activation [40]. 

Among these ICIs, anti-PD-1/PD-L1 monoclonal antibodies stand out, which bind to the inhibitory receptor PD-1 on tumor-reactive T cells and to PD-L1. However, in tumor cells, the PD-1/PD-L1 interaction is then disrupted to reactivate anti-tumor T-cell-mediated cellular cytotoxicity [88]. Examples of these previously described drugs are pembrolizumab and nivolumab [89,90,91].

### 5.14. Anti-TROP2 Antibodies

TROP2 (trophoblast cell surface antigen 2) is a transmembrane glycoprotein and intracellular transducer of calcium signals that may be overexpressed in squamous cell lung cancer, related to higher mortality. To neutralize this glycoprotein, monoclonal antibodies were created that recognize a single immunodominant epitope located between the globular and truncal regions of TROP2, and a great example of this drug class is sacituzumab govitecan [45].

## 6. How to Reverse Resistance to Therapeutic Antibodies and TKIS

### 6.1. Resistance to mAbs and TKIs

Resistance to target therapy can be developed by two mechanisms: (1) primary resistance or (2) acquired resistance, also known as on-target and off-target, respectively [4,92]. The first is related to mutations in the drug’s primary molecular target, leading to low or no response, while off-target resistance is related to responses in parallel signaling pathways or downstream of the target [4,92]. 

#### 6.1.1. Main Genetic Alterations

Numerous gene alterations have been identified that impact therapy selection and the testing of lung cancer specimens for these alterations are important for the identification of potentially efficacious targeted therapies as well as for avoiding therapies unlikely to provide clinical benefit. NCCN guidelines also highlight PD-1/PD-L1, although they indicated that PD-L1 expression can be elevated in patients with oncogenic driver mutations and that targeted therapy should take precedence over immune checkpoint inhibitors. Indeed, in the section titled “emerging biomarkers to identify novel therapies for patients with metastatic NSCLC”, they included high-level *MET* amplification as a genetic alteration (i.e., driver event) and indicated that available targeted agents with activity against driver events in lung cancer are capmatinib, tepotinib, and crizotinib [3,93]. NCCN guidelines indicate that, in general, the mutations/alterations described are seen in a non-overlapping fashion, although between 1% and 3% of NSCLC cases may harbor concurrent alterations [3,93].

**Figure 2:**
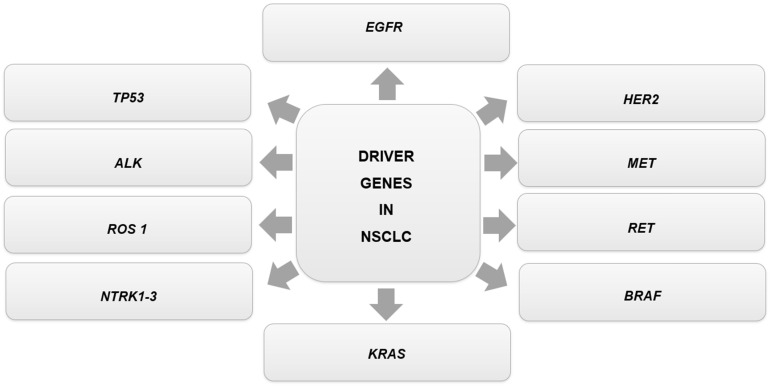
Driver genes in NSCLC. Driver genes can be classified as oncogenes and tumor suppressor genes and, in NSCLC, the main oncogenes are *EGFR*, *HER2*, *MET*, *RET*, *BRAF*, *KRAS*, *NTRK1-3*, *ROS 1*, and *ALK*. Oncogenes tend to hold mutations that activate proteins, which enhance tumor growth and cell proliferation, and are positively selected in cancer progression; therefore, targeted therapies focused on those genes are promising for the treatment of NSCLC. In contrast, tumor suppressor genes are essential for controlling cell division and replication of genetic material, avoiding imbalances, so they are usually deactivated in tumor selection and the main tumor suppressor gene in NSCLC is *TP53*, which is also responsible for the survival of several other histological tumor types.

NSCLC diagnosis is usually late, and targeted therapy presents itself as an option for patients in more advanced stages with driver mutations [42]. Oncogenic driver mutations are responsible for the beginning of tumor development and maintenance. They are essential to cell proliferation and tumor survival [42]. Among these alterations, the most frequent ones are related to the tyrosine kinase genes/receptors *EGFR*, *ALK*, *ROS1*, *MET*, *RET*, *NTRK 1-3*, and *HER2* [3]. There are other important driver genes in NSCLC, as shown below in Figure 2, as well as in Table 2, which summarizes oncogenic driver genes and their alterations with respect to drug resistance.

**Figure 3:**
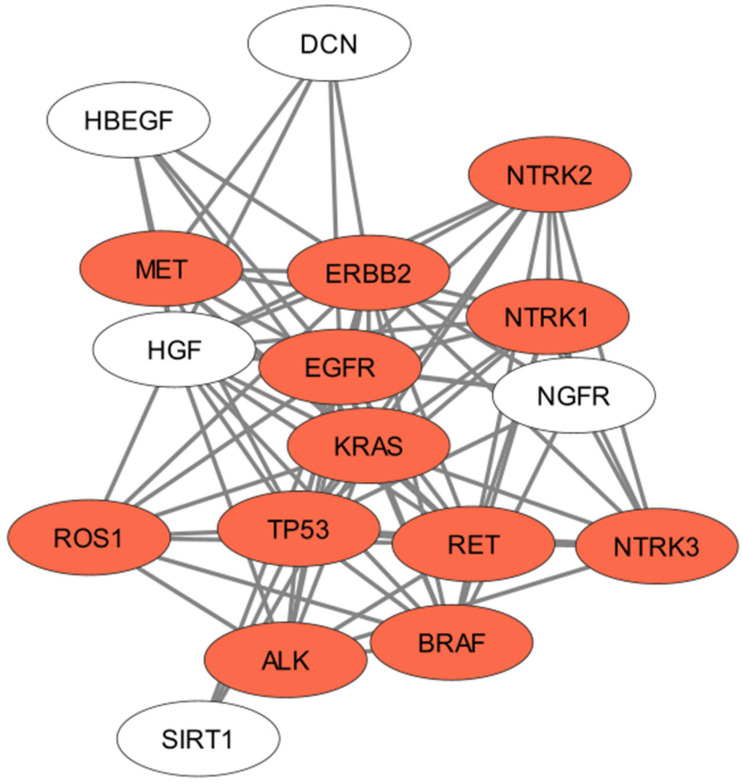
Protein–Protein Interaction Network associating proteins/driver genes in NSCLC. STRING version 12.0 (string-db.org) and Cytoscape version 3.10.1 (cytoscape.org) were used to create this representation. Evidence values were used for protein interaction (percentage values at an evidence level of around 40%) according to experimental, curation, text mining, and predictive computational data. Caption: Driver genes in red (RGB:251,106,74-FB6A4A).

To better demonstrate the interaction of all proteins/driver genes related to NSCLC, we created a Protein–Protein Interaction (PPIN) network, which can be visualized in Figure 3. *EGFR*, *KRAS*, *TP53*, *ALK*, *ROS* 1, *NTRK1-3*, *HER2*, *MET*, *RET*, and *BRAF* stand out as central points in the NSCLC PPIN, demonstrating their importance. From this Protein–Protein Interaction network created considering the relevant articles in Oncology and important driver genes, we suggest that these molecular targets should be studied together to understand how to reverse tumor resistance and how to better apply and combine novel sensitizing agents for NSCLC treatment.

The most prevalent molecular subtype in NSCLC is *EGFR*-mutant, with a prevalence of 15–20% in adenocarcinomas of patients in Caucasian populations and up to 50% in Asian populations [88,95]. As alternatives to kinase inhibitor treatment in NSCLC, drugs using ICIs have been developed to reverse the immune system evasion mechanisms acquired by tumor cells [96]. As previously described, some of the most used ICIs in NSCLC treatment are those that interfere with the PD-1/PD-L1 pathway [96]. *EGFR*-mutant patients generally have a low response to anti-PD-1/PD-L1 treatment, by mechanisms not yet well understood, but investigations suggest that this resistance is due to low PD-1 expression associated with low tumor mutational burden (TMB) [88]. Jia-Xin Li et al. (2019) define the tumor mutation burden as the total number of errors detected in the somatic gene coding, base substitution, gene insertion, or deletion per million bases, presenting itself as a biomarker that reflects the total number of mutations in tumor cells [96].

#### 6.1.2. Elucidating Epigenetics Alterations in Lung Cancer for Improved Therapeutic Interventions

Entire genome hypomethylation leads to instability and pathological activation of oncogenes. On the other hand, promoter hypermethylation can selectively inhibit gene expression, inhibiting tumor suppressor genes [4]. DNA hypermethylation profiles related to homeobox (*HOX*) genes and high mesenchyme-*HOX2* (*MEOX2*) gene expression are associated with drug resistance in lung cancer patients [4].

Li et al. (2013) showed a relationship between *EGFR* gene promoter DNA methylation in NSCLC cells and resistance to TKIs. The authors found that decitabine, a DNA methyltransferase inhibitor, could sensitize gefitinib response, resulting in inhibition of tumor cell growth and apoptosis, as well as decreased EGFR protein expression [4,97]. In another study conducted by Wen-Juan Liu et al. (2019), epigenetic alterations were shown to be associated with resistance to *EGFR* TKIs, being gradually induced during treatment [4]. To overcome this, a treatment combination of panobinostat/vorinostat with erlotinib. Histone deacetylation may promote the survival of *EGFR* TKIs-resistant cells and, when a deacetylation inhibitor (panobinostat/vorinostat) is combined with erlotinib, in vitro therapeutic effects are greater [4,98].

### 6.2. Exploring Alternatives Approaches to Reverse Resistance

NSCLC drug resistance has been addressed by several clinical trials seeking to minimize its effect [95]. Conventional therapies such as chemotherapy and radiotherapy cannot differentiate between tumor cells and healthy tissue, and normal cells are commonly damaged, resulting in numerous side effects [41]. These complications limit patients’ quality of life, compromising their ability to receive treatment and sometimes limiting the possibility of a favorable outcome. 

Targeted therapy acts against specific tumor molecular structures or abnormalities that are absent (or less abundant) in normal cells. Many of these agents have been introduced in clinical trials, and some have already been incorporated into clinical practice [3]. Recent studies point out that rapid and multilevel tumor gene expression epigenetic modifications are one of the most relevant causes of drug resistance and that the pattern of gene expression constantly evolves, resulting in the development of acquired resistance [4]. Thus, different immunotherapy-based methods, including monoclonal antibodies and TKIs, have sought a significant improvement in mortality in NSCLC patients. However, a large proportion of patients will experience acquired resistance to first-line chemotherapy, immunotherapy, or targeted therapy, requiring specific drug treatment [99].

#### 6.2.1. Overcoming Resistance Mechanisms in ALK+ NSCLC

The anaplastic lymphoma kinase positive (ALK+) NSCLC corresponds to 3–7% of all NSCLC diagnoses. ALK resistance mechanisms are complex, but several targeted therapy options for patients with this neoplasia are now available [100]. One of the first ALK TKIs that emerged for advanced ALK+ NSCLC treatment was crizotinib, approved by the Food and Drug Administration (FDA) in 2011, which demonstrated significant results, but eventually developed resistance mechanisms [101]. Researchers devising a therapeutic approach for patients with crizotinib resistance should consider several important factors when selecting the ALK TKI, including resistance profiles, systemic activity, central nervous system (CNS) activity, and patient safety [100].

In addition, lorlatinib is a third-generation ALK TKI that received an FDA breakthrough therapy designation in 2017 for patients whose cancer had progressed on prior ALK-directed therapy [102]. This drug has benefits against most known mutations in the ALK kinase domain, including the *ALK G1202R* mutation, which was found more frequently in patients with disease progression on second-generation ALK TKIs. Data from a recent study of lorlatinib showed significant clinical activity in patients with ALK+ NSCLC, many of whom had previously been treated and had CNS involvement [100].

#### 6.2.2. Combining PD-1/PD-L1 Inhibitors with Targeted Therapy

Targeted drug therapy against small cell lung cancer has a rapid effect, but over time, individuals may develop resistance. Clinical trials have evaluated the combination of immunotherapy and PD-1/PD-L1 blockade and targeted therapy in patients with *EGFR* mutant NSCLC [88].

The *EGFR* gene is the most common driver gene in NSCLC, and about half of Asian patients with NSCLC, especially those with lung adenocarcinoma, have mutations in the *EGFR* gene [96]. EGFR gene mutation is capable of positively regulating the expression of PD-L1 in tumor cells; *EGFR*-TKI can negatively regulate the expression of PD-L1 by inhibiting the nuclear factor-κB (NF-κB) signaling pathway [96]. Other studies have found that PD-1/L1 inhibitors can significantly reduce the viability of H1975 cells in vitro that are resistant to gefitinib, suggesting that combined immunotherapy with TKI may be an alternative for the treatment of patients with *EGFR* mutation in NSCLC, especially those who are resistant to *EGFR*-TKI [96]. 

On the other hand, in clinical trials, the combination of *EGFR* TKIs with PD-1/PD-L1 inhibitors did not demonstrate a favorable clinical efficacy in patients with *EGFR*-mutant NSCLC. Although the above clinical trial responses are still provisional, the optimal schedule, sequence, and dosage need to be carefully analyzed. In addition, the risk of toxicity for the patient needs to be evaluated in each study related to this combined therapy. Therefore, a comprehensive investigation into the mechanisms of action and the risks associated with drug combinations is essential [88].

A recent clinical study, CheckMate 012, used a relatively small sample size (n = 20) and analyzed the combination of nivolumab and different agents, including erlotinib, to treat NSCLC [41]. Nivolumab combined with erlotinib resulted in a clinical ORR of 19% and a 24-week PFS rate of 47%. Of the twenty patients with acquired resistance to erlotinib, three (15%) had partial tumor remission, and nine (45%) had stable disease. However, researchers found that there were four cases of treatment-related adverse reactions, mainly including an increase in aspartate aminotransferase (AST) and alanine aminotransferase (ALT) [96]. These data are still being studied, but they suggest that the nivolumab with erlotinib combination may favor sustained clinical improvement and safety for patients with *EGFR*-mutant NSCLC, especially those with TKI resistance [96].

#### 6.2.3. EGFR Therapy with Exon 20 Insertion Mutant Receptor

As mentioned previously, activating mutations of the *EGFR* gene are highly prevalent in NSCLC [103]. In clinical studies of this mutation pathway, the insertion of base pairs in exon 20 results in a constitutive activation of *EGFR*, which is different from the “classical” mutations that are more common in *EGFR* (L858R and deletions in exon 19) and associated with resistance to *EGFR* TKIs [104]. 

To overcome resistance to therapies targeting *EGFR* exon 20 insertion mutations, two targeted drugs are noteworthy and are being evaluated to meet this unmet clinical need: the small-molecule *EGFR* kinase inhibitor, mobocertinib, and the bio-specific anti-*EGFR*-*MET* antibody, amivantamab [95].

##### Mobocertinib

The irreversible covalent *EGFR* and *HER2* kinase inhibitor, mobocertinib, has activity against *EGFR* exon 20 insertion. Clinical studies were conducted to analyze the efficacy of this drug (160 mg of mobocertinib, administered once daily) in 114 patients with *EGFR* exon 20 insertion and prior platinum-based therapy, resulting in an ORR of 28% and PFS of 7.3 months in these patients, which was superior to poziotinib, another irreversible covalent kinase inhibitor against *EGFR* exon 20 insertion mutations [105].

##### Amivantamab

Amivantamab is a human monoclonal IgG1 antibody that targets both *EGFR* and *MET*, which are involved in driving tumor growth in lung cancer and activation of the *MET* pathway through the positive regulation of *MET* expression. This has been frequently described as a mechanism of resistance to *EGFR* kinase inhibitors [106]. 

Amivantamab monotherapy was evaluated in a cohort of *EGFR* exon 20 insertion mutant NSCLC patients who had progressed on platinum-based chemotherapy and included an efficacy population (81 patients) and a safety population (114 patients). In the efficacy cohort (n = 81), an ORR of 40% was observed, including three confirmed complete responses (CR) and 29 partial responses (PR), while 39 patients had stable disease (SD). In this cohort, the median PFS was 8.3 months [95]. Trials with this drug are still being evaluated to establish combinations with other first-line therapies for NSCLC, but it already shows good clinical activity against this class of mutations [106]. Additionally, the dual targeting of *MET* may provide this drug an advantage over other *EGFR* inhibitor monotherapy strategies, avoiding the manifestation of resistance through *MET* activation and thus resulting in a more permanent solution [95].

## 7. Novel Biomarkers for NSCLC

### 7.1. Epigenetics as a Source of Novel Biomarkers for Lung Cancer

Epigenetic alterations are promising sources of biomarkers in several types of cancers, especially lung cancer, where miRNAs, DNA methylation, and IncRNAs have been widely studied [106,107,108]. These molecular markers have shown a high potential to improve diagnostic accuracy, identify tumor subtypes, predict disease progression, and direct more effective therapies, boosting efforts to search for more personalized strategies [109,110].

Tests with miR-34, for example, have detected lung cancer in 80% of high-risk asymptomatic smokers. Interestingly, the low expression of these miRNAs was related to higher chances of relapse [111], while the low expression of miR-30a, miR-107, miR-138, miR-204, miR-32, miR-148b, miR-145, miR-224, miR-200c, miR-125b, and miR-375 predicted poor clinical outcome and events such as lymph node metastasis and tumor enlargement [111]. Likewise, miRNA biomarkers are also relevant in the response to treatments such as immunotherapy and targeted drugs. In patients with advanced NSCLC, three miRNAs (miR-21, miR-27a, and miR-218) were overexpressed in the treatment-resistant group compared to the sensitive group [106]. This underscores the importance of miRNA biomarkers in identifying patients who may best respond to specific therapies, thereby directing treatment toward greater efficacy [111,112].

DNA methylation has also been suggested as a promising biomarker for lung cancer diagnosis [113]. Research conducted by Zhang et al. [114] investigated methylation profiles in lung cancer patients. Nine genes (*APC*, *CDH13*, *KLK10*, *DLEC1*, *RASSF1A*, *EFEMP1*, *SFRP1*, *RARβ*, and p16 (*INK4A*) showed a higher frequency of methylation in NSCLC when compared to healthy tissues. Vrba et al. [115] clinically evaluated a set of ten DNA methylation biomarkers (*LINC01158*, *CCDC181*, *PRKCB*, *TBR1*, *ZNF781*, *MARCH11*, *VWC2*, *SLC9A3*, *HOXA7*) in NSCLC patients, showing significant differences in the methylation pattern of these biomarkers between NSCLC patients and healthy subjects. In another study, Zhang et al. [110], mapped 5-hydroxymethylcytosine (5hmC) signatures in the circulating DNA of patients with NSCLC. A significant gain of 5hmC was observed in the promoter regions in blood samples from patients with the disease. Furthermore, through high-precision machine learning, six potential biomarkers were identified (*SIPA1L2*, *RSPO3*, *LDB2*, *ZNF679*, *AP 001604.3*, *RP1-137K24.1*). These findings show the potential of these epigenetic biomarkers as important tools in the diagnosis and management of lung cancer, as shown in Table 3. However, epigenetic biomarkers present challenges, including the panel inconsistency used in different studies, making it difficult to compare and define an ideal diagnostic model. Establishing a promising model requires large-scale clinical trials for validation, thus highlighting the urgency of these studies [106,115].

### 7.2. Single-Cell Analysis: A Recent and Promising Strategy for Biomarker Discovery

As the lung is a complex and molecularly heterogeneous organ, studies on the composition of NSCLC tend to be limited when performed by conventional analysis, mainly due to the use of millions of cells in their study. Therefore, the results obtained usually only show average molecular changes, veiling tumor heterogeneity [137].

On that account, single-cell analysis emerges as an innovation in liquid biopsy research, allowing the detailed molecular characterization of NSCLC, being revolutionary in the transcriptome sequencing of circulating tumor cells as well as immune cells associated with the pathological process [137,138]. This technology allows the identification of heterogeneous subpopulations in terms of histological types, tracking their behavior and evolution, as well as evaluating the current molecular state of the cancer and classifying new biomarkers [138].

#### 7.2.1. Biomarkers and Single-Cell Analysis

Single-cell analysis allows for the assessment of a tumor and its microenvironment with high precision, including parameters such as surface protein expression, transcriptomic alterations, and others. Thus, these can be integrated for the study of immunotherapeutic responses and, above all, in the identification of future biomarkers [139].

In this area, studies conducted by mass cytometry enabled the identification and functional characterization of various cell subtypes at the protein level. As samples in this technique are stained with antibodies conjugated to heavy metal isotopes, spectral overlap observed with flow cytometry is avoided [139].

In line with this, Datar et al. [140] phenotyped infiltrated leukocytes from 20 resected primary NSCLCs to assess the biological implications of the expression of three negative immune checkpoints: PD-1, LAG-3, and TIM-3. A panel of 35 markers enabled the monitoring of nine different immune populations: CD8+ T cells, CD4+ T cells, regulatory T cells (Tregs), natural killer (NK) cells, B cells, granulocytes, macrophages, and dendritic cells. Their results suggested that LAG-3 might be involved in immunotherapy resistance and could be targeted to enhance the response to immune checkpoint inhibitors [139,140].

In parallel, Tay et al. [136] analyzed blood samples from NSCLC patients treated with nivolumab, and their findings revealed that Treg lymphocytes were more prevalent in the blood of non-responding patients, while responders showed higher frequencies of CD62Llow CD8+ and CD4+ T cells [136,139].

In summary, Prazanowska et al. [141] integrated seven independent single-cell RNA sequencing datasets (scRNAseq) and provided a library with single-cell information from 224,611 primary NSCLC tumor cells, organizing the results into nine groups of cells related to immunological processes. Among these, it is worth mentioning macrophages with a high expression of mitochondrial genes and genes encoding ribosomal proteins are related to greater damage and oxidative stress. This information allowed the creation of a timeline of T cell exhaustion, a process that demands an orchestrated activation—patients with overexpression of initiator genes, such as *DUSP1*, tend to have a better prognosis when compared to those more expressed at the end of the process, such as *TXNRD1* [141].

Li et al. [137] explored the cellular diversity in lung tumor tissues by analyzing epithelial cells from normal and affected tissues. Cancer cells simultaneously expressing classic biomarkers of up to three histological types of NSCLC were identified: adenocarcinoma, squamous cell carcinoma, and neuroendocrine tumors. Such a cell lineage, classified as mixed by the researchers, was correlated with cases of worse prognosis, indicating that such a combination may constitute a biomarker of malignancy. Furthermore, the study of this subpopulation revealed a new therapeutic target, the *AKR1B1* gene, which is overexpressed in cells with greater tumor plasticity, related to cell proliferation processes and evasion of apoptosis [137].

Peng et al. [142] developed a tool based on multiplex immunofluorescence images capable of analyzing the intercellular interaction networks in the tumor microenvironment, revealing that neoplastic and immune cell architecture and behavior are fundamental for prognosis, in a way that greater spatial proximity between T and B lymphocytes and N1-type neutrophils was correlated with a better outcome, since such organization facilitates immune response antitumor action. However, Treg cells close to neutrophils were associated with less leukocyte migration, immunosuppression, and angiogenesis, contributing to tumor growth and infiltration [142].

#### 7.2.2. RNA-Seq and Single-Cell Analysis

RNA sequencing (RNA-seq) can access not only information about the cell type but also its relation to the tumor microenvironment. Pang et al. [143] integrated two large-scale RNA sequencing datasets to illustrate the complex cellular communication network and characterized four differentiation states and genes related to neutrophil differentiation. Their results were able to establish a model that can be used to predict patient prognostic performance and immunotherapeutic efficacy in NSCLC patients [139,143].

Lau et al. [144] demonstrated that NSCLC cells expressed transcripts of human leukocyte antigen (HLA)-II, primarily HLA-DRB1. Another significant finding was the identification of a group of CD4+ cytotoxic GZMB T cells that expressed high levels of PDCD1 and CTLA4, suggesting that this population could be involved in antitumor response post-immunotherapy [139,144].

In Sultana et al. [145], they used single-cell-RNA sequencing (scRNA-seq) to determine transcriptional profiles and biomarkers related to NSCLC gene expression. The identification of tumor heterogeneity in tissues and the evaluation of gene expression levels allowed the study of associated pathways, such as those related to miRNAs and growth factors, relevant for determining NSCLC diagnosis and prognosis. Finally, of the 12 main genes identified, the technique detected two new genes related to lung cancer evolution: *TYROBP* and *RPLP1* [145].

#### 7.2.3. Bioinformatics and Single-Cell Analysis

Single-cell-based methods are able to determine unique molecular identifiers in different tumors. Bioinformatics tools can create databases and libraries for online consultation, and assembling panels with key genes is useful to determine the transcriptional profiles of circulating tumor cells (CTCs), correlating to certain expression patterns with prognosis and metastasis [138].

CTC is a well-established liquid biopsy CellSearch system, which is an immunomagnetic purification method. CTCs serve as a potential diagnostic, prognostic, and predictive biomarker in metastatic cancers such as lung, breast, colorectal, and prostate. Wang et al. [146] analyzed the alteration of baseline CTC levels during platinum chemotherapy in previously untreated stage III or IV NSCLC patients. Their results suggested that the persistent presence of CTCs during treatment means a poor prognosis and therapy resistance in advanced NSCLC [146].

Brockley et al. [138] mention several bioinformatics tools and their relevance for processing single-cell sequencing data, encouraging the assembly of databases for consultation and the pre-processing of raw data, quality control, and gene counting, increasing the accuracy and effectiveness of the diagnosis and treatment of lung cancer. The Ginkgo online platform is capable of identifying cell variants through sequencing codes, such as a “barcode”, so that it is possible to study tumor heterogeneity by liquid biopsy cell phylogenetics [138].

Monocle is a software able to determine cellular development networks and changes in gene expression, while programs such as Scanorama identify cells with similar transcriptional profiles and establish correlations in order to classify them. Finally, other tools, such as OncoNEM and SCITE, are innovative in tracing the evolutionary trajectory of cancers from single-cell information, indicating a promising future for highly technological and minimally invasive treatments [138].

### 7.3. Multiple Cellular Stress Responses in Lung Cancer: A Source of Novel Biomarkers?

Years of NSCLC oncological research have provided countless insights, yet little progress has been observed in treatment, except for extending survival by a few months/years [147]. This is probably because many biological processes and mechanisms that underlie tumor cellular responses to environmental and intrinsic stressors are often overlooked or misinterpreted (such as polyploidization, multinucleation, micronucleation, premature senescence, apoptotic reversal, cell fusion, cell-cell structures, syncytial formations, and others) [148,149,150,151,152,153].

NSCLC emerges as a tumor highly affected by therapeutic stressors, capable of inducing reversible polyploidization and premature senescence through evolutionarily conserved mechanisms to evade genotoxic insults from radiotherapy, chemotherapy, or other treatments, being a mechanistic compilation with potential new sources of biomarkers and prognostic factors [154,155].

As a consequence, what emerges is a “survival kit” for NSCLC, enabling the generation of giant cancerous polyploid cells (PGCCs), polyploid circulating tumor cells, polyploid senescent cells, cancer-associated macrophage-like giants (CAMLs), therapy-resistant cancer stem cells, and rapidly proliferating nearly diploid or aneuploid cells, all of which contribute to a worse prognosis and enhanced progression of NSCLC [154,155,156,157,158].

Understanding NSCLC responses becomes crucial in providing efficient tumor biomarkers. Table 4 highlights a bibliographic compilation of potential stress biomarkers for NSCLC.

## 8. Conclusions

Over the past two decades, there has been a significant increase in the amount of information available regarding the molecular biology of cancer, including Oncogenetics. One of the biggest challenges in Oncology is to effectively translate this information into clinical advances and improve patient care. Tumor development is characterized by complex mechanisms that directly influence patient prognosis, especially given the extensive heterogeneity of NSCLC, which is characterized by distinct genetic abnormalities even within the same tumor and between primary and metastatic tumors. The treatment landscape for this cancer subtype has undergone significant changes since the introduction of molecular targeted therapy, which has demonstrated superior benefits compared to established oncological treatments, such as chemotherapy, with fewer adverse effects and improved survival rates. 

In this paper we presented a historical guide of NSCLC biomarkers, from classic to innovative approaches, showing the biomarkers identified by epigenetic information, mainly non-coding RNAs acting at the regulatory level in NSCLC. Furthermore, Single-cell approaches are potential sources of new biomarkers that encompass the heterogeneity and complexity of NSCLC. Stress biomarkers are novel in this scenario, showing that tumor cells can vary their ploidy, helping tumor adaptation and aggressiveness. 

Resistance to immunotherapy and tyrosine kinase inhibitors (TKIs) remains a challenge in advanced disease. Currently, there is a trend toward personalized medicine and the use of more sophisticated and complex patient information to enhance treatment specificity and efficiency. Therefore, the present and future of NSCLC treatment are based on genotyping and prognostic markers, which enable a combination of drugs with precise targeting capabilities to eradicate tumors and improve patient quality of life. 

## Figures and Tables

**Table 1:** Clinical trials table of monoclonal antibodies (MAbs) and tyrosine kinase inhibitors (TKIs) used in the treatment of lung cancer. The names of the drugs are described, as well as the class to which these inhibitors belong and the respective study number in https://clinicaltrials.gov, accessed on 20 July 2023.

Drug Name	Class	Study/NCT * Name
Nivolumab	Anti PD-1	CheckMate-012 (NCT01454102) CheckMate 227 (NCT02477826)
Pembrolizumab	Anti PD-1	KEYNOTE-001 (NCT01295827)
Atezolizumab	Anti PD-1	POPLAR (NCT01903993) OAK (NCT02008227)
Durvalumab	Anti PD-1	PACIFIC (NCT02125461)
Sacituzumab Govitecan	Anti-TROP2	TROPION-PanTumor01 (NCT03401385)
DS-102	Anti-TROP2	TROPION-PanTumor01 (NCT03401385)
Erlotinib	TKI EGFR inhibitor	EUTARC (NCT00446225)
Dacomitinib	TKI, ErbB/HER inhibitor	ARCHER1050 (NCT01774721)
Osimertinib	TKI, EGFR mutation inhibitor	FLAURA (NCT02296125)
Crizotinib	ALK inhibitor	NCT01154140
Alectinib	ALK inhibitor	ALEX (NCT02075840)
Brigatinib	ALK inhibitor	NCT02737501
Certinib	ALK inhibitor	NCT01828112
Lorlatinib	ALK inhibitor	NCT01970865
Trametinib	MEK inhibitor	NCT01336634
Selumetinib	MEK inhibitor	SELECT-2 (NCT01750281)

* NCT—National Clinical Trial number, also called the ClinicalTrials.gov Identifier (accessed on 20 July 2023).

**Table 2:** Oncogenic driver genes and their drug resistance alterations.

Oncogenic Driver Genes	Comments	Genetic Alteration	Targeted Therapies for the Genetic Alteration in NSCLC According to NCCN Guidelines Version 3.2023	References
*EGFR*	The alterations are related to the PIK3/AKT/mTOR and RAS/RAF/MEK pathways, which induce anti-apoptotic activity, cell proliferation, and promote tumor growth and survival. These abnormalities consist of gene mutation, gene amplification, and protein overexpression.	*EGFR* Exon 19 Deletion or L858R	First-line therapy: afatinib; erlotinib; dacomitinib; gefitinib; osimertinib; erlotinib + ramucirumab; erlotinib + bevacizumab (nonsquamous). Subsequent therapy: osimertinib.	[4,42,94]
		*EGFR* S768I, L861Q and/or G719X	First-line therapy: afatinib; erlotinib; dacomitinib; gefitinib; osimertinib. Subsequent therapy: osimertinib.	
		*EGFR* Exon 20 Insertion Mutation	Subsequent therapy: amivantamab-vmjw; mobocertinib.	
*ALK*	Most *ALK*-positive patients carry the *ALK*-fusion gene 4 *(EML4)-ALK* rearrangement. This fusion gene encodes a protein related to cell proliferation, differentiation, and inhibition of apoptosis.	*ALK* Rearrangement Positive	First-line therapy: alectinib; brigatinib; ceritinib; crizotinib; lorlatinib. Subsequent therapy: alectinib; brigatinib; ceritinib; lorlatinib.	[4,42,94]
*ROS1*	The ROS1 kinase domain can fuse with differentiation cluster 74 (CD74), leading to its kinase activity. The changes are related to rearrangements in the *ROS1* gene.	*ROS1* Rearrangement Positive	First-line therapy: ceritinib; crizotinib; entrectinib. Subsequent therapy: lorlatinib; entrectinib.	[4,42]
*NTRK1-3*	The *NTRK1*, *NTRK2*, and *NTRK3* genes encode proteins that act as growth factor receptors in the nervous system during normal physiology. The alterations are related to oncogenic fusions of the *NTRK* genes.	*NTRK1/2/3* Gene Fusion Positive	Larotrectinib; entrectinib.	[4,42,94]
*MET*	The alterations relate to amino acid substitutions in Y1003 or mutations/deletions in METex14 (mutation of *MET* exon 14) or its lateral introns.	*MET* Exon 14 Skipping Mutation	Capmatinib; crizotinib; tepotinib.	[4,42]
*RET*	The alterations are related to gene rearrangements involving *RET*, resulting in dysregulation and inappropriate signaling through the RET kinase domain.	*RET* Rearrangement Positive	Selpercatinib; pralsetinib; cabozantinib.	[4,42,94]
*HER2*	A total of 96% of the alterations are kinase-activating exon 20 insertion mutations.	*HER2* Mutation Positive	Subsequent therapy: fam-trastuzumab deruxtecan-nxki; ado-trastuzumab emtansine.	[4,42]

Subtitle: *ALK*—anaplastic lymphoma kinase, *BRAF*—proto-oncogene B-Raf, *EGFR*—epidermal growth factor receptor, *HER2*—human epidermal growth factor receptor 2, *MET*—mesenchymal-epithelial transition factor, NCCN—National Comprehensive Cancer Network, NSCLC—non-small cell lung cancer, *NTRK 1-3*—neurotrophic tyrosine kinase type 1-3, *RET*—*RET* proto-oncogene, *ROS1*—*ROS* proto-oncogene.

**Table 3:** Potential Epigenetic Biomarkers and Their Role in NSCLC.

Epigenetic Modification	Biomarker	Regulation in NSCLC	References
DNA methylation	*DNMT1*, *MGMT*, *DAPK*, *RASSF1a*, *CDKN2A*, *APC*, *CHD13*, *KLK10*, *DLEC1*, *AGTR1*, *GALR1*, *SLC5A8*, *NTSR1*, *SULF2.*	Hypermethylation	[109,113,114,116,117,118,119]
	*ZMYND10*	Hypomethylation	[109,118]
	*PITX2*, *SHOX2*	Methylation	[120]
Histone Modification	*H4K5/H4K8*	Hyperacetylation	[109,118]
	*H4K12/H4K16*	Hypoacetylation	
	*H4K20me3*	Loss trimethylation	
miRNAs	*let-7c*, *miR-138*, *miR-145*, *miR-183*, *miR-29*, *miR-34a*, *miR-34c-3p*, *miR-101-3p*, *miR-129*, *miR-200b*, *miR-212*, *miR-218*, *miR-449a e miR-451*	Downregulated	[121,122,123,124,125]
	*miR-126*, *miR-21*, *miRs-34a miR-19*, *mi-150 e miR-141*, *miR-124*, *miR-132*, *miR-155*, *miR-331-5p e miR-483-5p*	Upregulated	[111,122,126,127,128]
	*miR-30a*, *miR-107*, *miR-138*, *miR-204*, *miR-32*, *miR-148b*, *miR-145*, *miR-224*, *miR-200c*, *miR-125b e miR-375*, *miR-23b-3p*	Downregulated	[106,126,129]
	*miR-21/155*, *miR-25*, *miR-31*, *miR-221/222*, *miR-224*, *miR-191*, *miR-494*, *miR-19a and miR-346*, *miR-10b-5p*	Upregulated	[122,129,130]
lncRNA	*MCM3AP-AS1*, *TP53TG1*	Downregulated	[131,132]
	*RP11-397D12.4*, *AC007403.1*, *ERICH1-AS1*, *SPRY4-IT1*, *ANRIL and NEAT1*	Upregulated	[133,134]
	*GAS5*; *HAGLR*, *ADAMTS9-AS2*, *TP73-AS1*, *LINC00261 e LINC00312*	Downregulated	[131,135]
	*TBILA*, *AGAP2-AS1*, *LINC00673*, *LOC730101*	Upregulated	[131,136]

**Table 4:** Potential Stress Biomarkers and for NSCLC.

Potential Biomarkers/Factors	Description	References
p16INK4A, p53, p21, CDK1 and survivin	• High expressions of specific genes are observed after radiotherapy in NSCLC;	[155,156,157,158]
	• Lung cancer cells lacking p53 have the ability to evade chemotherapy-induced senescence;	
	• CDK1 is a potential biomarker for the transition between senescence and the repopulation of cancer cells from giant polyploid cancer cells;	
	• Phenotypic characteristics of NSCLC lineages, A549 (wild-type *TP53*) and H1299 (*TP53* deficient), as well as their surviving descendants after multifractionated X-ray irradiation, exhibit a strong association with p53 as a biomarker for the formation of multinucleated giant cancer cells.	
Giant CAMLs	• Giant CAMLs as a potential peripheral blood biomarker for NSCLC progression due to their relationship with metastatic disease and worse survival, despite the use of maintenance immunotherapy.	[159]
CDH1	• CDH1 as a potential new drug target, and its hypermethylation can be reversed through demethylation, being used in lung cancer, which may present a possible relationship with the stress response mechanisms in NSCLC.	[160]
Aurora kinase A and B, JAK2, SRC, and histone H3	• Resistance to EGFR TKIs in NSCLC is frequently associated with activation of AURKB and increased levels of histone H3 phosphorylation;	[161,162]
	• Reversine as an anticancer agent in human NSCLC and influences potential cell cycle biomarkers associated with polyploidy such as Aurora kinase A and B, JAK2, and SRC.	
Staurosporine	• Association between staurosporine (potential biomarker) and PGCCs (giant cancer cells) and features of polyploid and multinucleated growth in lung cancer cell lines.	[163]
Mitotic microtubule polymerization	• Mitotic microtubule polymerization as a critical hallmark of NSCLC polyploidization after vinorelbine treatment, inducing prolonged accumulation in the G2/M phase after radiotherapy.	[164]
Vimentin and N-cadherin	• Inhibition of vimentin and N-cadherin in the face of the stressful effect of quercetin acts on the main elements of the cytoskeleton (microfilaments, microtubules, and intermediate filaments) but also associates with mitotic catastrophe, cytokinesis failure, induced G2/M arrest, polyploidy, increased cell size, and multinucleation in NSCLC.	[165]
FHIT	• In over 90% of lung tumors, FHIT exhibits loss of heterozygosity, and in advanced cases, it shows promoter methylation;	[166]
	• FHIT stands out as a key biomarker for understanding the bridge between macroevolution and microevolution in lung cancer;	
	• Abnormal FHIT expression promotes genomic instability, leading to increased aneuploid chromosomes, single-stranded DNA, and induced genetic mutations driving microevolution.	
Cyclin b1 e CDC2	• Proliferation and apoptosis characteristics of the docetaxel-induced polyploid NSCLC cellular model, as well as the potential role of polyploid tumor cells in chemotherapy resistance and tumor recurrence. Increased expression of anti-apoptotic proteins (such as bcl-2, pbcl-2, and bcl-xl) and survival proteins (such as survivin), along with the inhibition of cyclin B1/cdc2 complex activity in A549 cells, leading to a G2/M cell cycle arrest, and generation of polyploid tumor cells.	[167,168]
CD8+ and SBS3	• Evolution of the immune profile from primary tumors to distant and local metastases in NSCLC revealed that the level of CD8+ T cells was lower in polyploid samples than in diploid samples. Furthermore, the SBS3 signature, closely associated with genomic instability, exhibited a significantly higher proportion in polyploid metastases, highlighting it as a potential biomarker of tumor evolution.	[169]
ASAH1 and L858R	• Inhibition of acid sphingolipid enzyme ceramidase (ASAH1) activity as a complementary action in combination with cisplatin against NSCLC, favoring the reduction in aneuploid offspring resulting from the depolyploidization process of PGCCs;	[170,171,172,173]
	• Unlike other EGFR mutations, L858R needs dimerization that is inhibited by cetuximab, reducing the viability of cells expressing L858R-EGFR and blocking the FOXM1-aurora survival pathway, whereas other mutants show no responses;	
	• Cetuximab completely prevents relapses of L858R+ tumors, differently to TKI-treated patient-derived xenografts, which relapse post osimertinib treatment. Osimertinib’s lower efficacy is associated with the induction of mutagenic reactive oxygen species, while cetuximab downregulates adaptive survival pathways (HER2).	

## Data Availability

Not applicable.
